# Targeting NF-kappa B/proinflammatory cytokines/ TGF-β/ TIMP-1 pathway by crocin enhances recovery from hepatic fibrosis in rats

**DOI:** 10.1038/s41598-025-32672-w

**Published:** 2026-01-14

**Authors:** Memy H. Hassan, Samir A. Salama, Raed S. Ismail

**Affiliations:** 1 Department of Pharmacology and Toxicology, Faculty of Pharmacy (Boys), Al-Azhar University, Nasr City, Cairo, 11651 Egypt; 2 Division of Biochemistry, Department of Pharmacology, College of Pharmacy, Taif University, P.O. Box 11099, Taif City, 21944 Kingdom of Saudi Arabia

**Keywords:** Liver Fibrosis, CCl₄, Crocin, Inflammatory Cytokines, Extracellular Matrix, Oxidative Stress, Biochemistry, Diseases, Gastroenterology, Medical research

## Abstract

**Background:**

Liver fibrosis is a dynamic and potentially reversible process until irreversible structural changes occur. This study evaluated the curative effect of crocin on carbon tetrachloride (CCl₄)-induced hepatic fibrosis in rats and explored the underlying mechanisms.

**Methods:**

Thirty male rats were allocated into three groups: a control group treated subcutaneously (SC) with corn oil for 8 weeks followed by intraperitoneal (IP) saline for 2 weeks; a spontaneous recovery group (CCl₄-SC for 8 weeks followed by saline IP for 2 weeks); and a crocin recovery group (CCl₄-SC for 8 weeks followed by crocin 100 mg/kg/day IP for 2 weeks). Liver function tests, fibrosis biomarkers, collagen deposition, inflammatory mediators, oxidative stress indices, and the gene expression of collagen I and α-SMA were assessed using ELISA, spectrophotometry, or qRT-PCR.

**Results:**

Crocin significantly improved liver function and reduced fibrosis markers (hyaluronic acid, laminin, PCIII, hydroxyproline, TGF-β, TIMP-1). Furthermore, it downregulated collagen I and α-SMA expression and suppressed NF-κB mediated inflammatory cytokines (TNF-α, IL-1β, NO). It also enhanced antioxidant defenses (GSH, SOD, catalase, GSH-Px) compared with the spontaneous recovery group.

**Conclusion:**

Crocin exerts a promising curative effect against CCl₄-induced hepatic fibrosis in rats by suppressing NF-κB driven inflammation and profibrogenic mediators, thereby limiting collagen deposition.

## Background

Advanced liver fibrosis is the common final pathway for the majority of chronic liver injuries, resulting in cirrhosis and can subsequently lead to hepatic failure and hepatocellular carcinoma ^1^. Fibrosis is a dynamic process and remains reversible before progressive architectural changes occur in the liver ^2,3^.

Liver fibrosis is identified by the accumulation of extracellular matrix (ECM) proteins secreted by activated hepatic stellate cells (HSCs). HSC proliferation and differentiation into myofibroblast-like cells are involved in the development of liver fibrosis ^4^. Accumulation of ECM proteins distort liver structure through the formation of fibrotic scars and the subsequent development of nodules of regenerating hepatocytes leads to cirrhosis. Thereupon, cirrhosis induces liver insufficiency and portal hypertension, respectively ^5,6^.

Chronic liver injury and repair ensuing CCl_4_ exposure release a large amount of inflammatory mediators from HSCs e.g. nuclear factor kappa-light-chain-enhancer of activated B cells (NF-κB), tumor necrosis factor alpha (TNF-α) and transforming growth factor (TGF-β) ^7–9^. In addition, cytokines including transforming growth factor-β (TGF-β), tumor necrosis factor-alpha (TNF-α) and interleukin-1β (IL-1β) promote HSCs trans-differentiation, the main source of ECM in the development of liver fibrosis ^10,11^. Furthermore, these proinflammatory cytokines are partially controlled by the NF-κB, which is primed by oxidative stress and signaling pathway of many cytokines including IL-1β and TNF-α, generating extra waves of inflammation and ECM accumulation ^12^.

Oxidative stress plays a pivotal role in provoking liver damage and consequent fibrogenesis. Hepatocytes undergo necrosis and apoptosis as consequence of oxidative protein, lipid, and DNA damage generating reactive oxygen species (ROS), which also intensifies the inflammatory response and starts the fibrosis process. Additionally, ROS stimulate Kupffer cells and other inflammatory cells to produce a profibrogenic mediators ^6,13^.

Hepatic stellate cell (HSC) activation is the central event in the development and progression of liver fibrosis ^4^. In response to chronic injury, quiescent HSCs transform into proliferative, contractile, and ECM-producing myofibroblast-like cells, leading to excessive collagen deposition and architectural distortion of liver tissue ^5,6^. Activated HSCs also secrete multiple profibrogenic mediators, including TGF-β and TIMP-1, which further sustain ECM accumulation and inhibit matrix degradation ^5,6^.

Thus, it is conceivable that intercepting HSC activation and/or its signaling pathway consequences would be a perfect therapeutic target for reversing hepatic fibrosis. Therefore, administering an antioxidant agent with anti-inflammatory effect could be beneficial in preventing liver cirrhosis via speeding up recovery of liver from fibrosis.

Crocin is the main active constituent of saffron with pleiotropic pharmacological activities including antioxidant, anti-tumor, radical scavenging, and genoprotective activities ^14–16^. Moreover, our earlier research has indicated that crocin can prevent CCl4-induced liver injury by, at least in part, attenuation of oxidative stress and inflammation ^17^. As a continuation to our previous work, this study looked into whether crocin can cure CCl4-induced liver fibrosis and furthermore the aim was extended to establish the possible underlying mechanism.

## Materials and methods

### Materials

Carbon tetrachloride (CCl₄), crocin, and corn oil were obtained from Sigma-Aldrich (St. Louis, MO, USA). Crocin was freshly dissolved in sterile normal saline before use.

### Animals

Male Sprague–Dawley rats (200–250 g) were housed in the animal facility of the Faculty of Pharmacy, Al-Azhar University, Cairo, Egypt under standard laboratory conditions (12-h light/dark cycle, 25 ± 1 °C, 55% relative humidity) with free access to water and chow. All experimental procedures were approved by the Ethics Committee of Taif University, KSA, and Al-Azhar University, Egypt (Protocol no. TUCDREC/2,016,012 Hassan) and were conducted in compliance with ARRIVE guidelines.

### Experimental design

After acclimatization, 30 rats were randomly divided into three groups (n = 10):

- **Control**: corn oil SC twice weekly for 8 weeks, followed by saline IP for 2 weeks.
- **Spontaneous Recovery group (SRG)**: CCl₄ in corn oil (50% v/v, 2 mL/kg, SC, twice weekly for 8 weeks), then saline IP for 2 weeks.
- **Crocin Recovery group (CRG)**: same CCl₄ regimen, then crocin (100 mg/kg/day, IP) for 2 weeks.

The crocin dose was selected based on previous dose response studies ^14,16,17^.

Rats were monitored daily and weighed weekly. At week 10, animals were anesthetized with isoflurane (3% induction, 1.5–2% maintenance) and euthanized by cervical decapitation.

### Assessment of liver health

Relative liver weight was calculated using the formula:

$$\text{Liver weight ratio }\left(\mathrm{\%}\right)=\frac{\text{Liver weight }(\mathrm{g})}{\text{Body weight }(\mathrm{g})}\text{ X }100$$

Serum alanine aminotransferase (ALT), aspartate aminotransferase (AST), alkaline phosphatase (ALP), and bilirubin were measured using commercial kits (Spectrum Diagnostics, Cairo, Egypt) with an automated analyzer (Mindray BS-200, Shenzhen, China)^20^.

### Evaluation of liver fibrosis

#### Serum fibrosis biomarkers

Levels of hyaluronic acid, type III procollagen (PCIII), and laminin were measured using a radioimmunoassay kit (Cat. No. h141; Shanghai Haiyan Pharmaceutical Technology, China). In addition, PCIII and laminin were determined using ELISA kits (Cat. No. MBS765694; BioSource, Camarillo, CA, USA, and Cat. No. AB119573; Abcam, Cambridge, UK, respectively).

#### Assessment of hydroxyproline level in rat liver

Hepatic collagen content was assessed by measuring hydroxyproline levels in liver tissue ^18^. Liver samples were hydrolyzed, treated with chloramine T (2.5 mM), then with p-dimethylaminobenzaldehyde (410 mM), and incubated at 60 °C for 30 min. Absorbance was recorded at 560 nm using a standard curve. Results were normalized to tissue weight, with each sample analyzed in triplicate, and data expressed as group mean ± SEM.

#### Tissue homogenization

Liver tissue (100 mg) was homogenized in ice-cold RIPA buffer using a glass–Teflon homogenizer. The homogenates were centrifuged at 10,000 g for 15 min at 4 °C, and the supernatants were collected for subsequent biochemical and ELISA analyses.

#### Assessment of transforming growth factor-beta (TGF- β) and tissue inhibitor of matrix metalloprotease-1 (TIMP-1) level in rat liver

Liver homogenates were used to determine TGF-β levels with a commercial ELISA kit (Biosource, Camarillo, CA, USA; Cat. No. MBS824788) and TIMP-1 levels with an ELISA kit (R&D Systems, Minneapolis, MN, USA; Cat. No. RTM100). All values were normalized to the corresponding total protein content.

### Evaluation of mRNA expression for collagen I and alpha smooth muscle actin

Total RNA was extracted from liver tissues using TRIzol Reagent (Invitrogen Life Technologies, Carlsbad, CA, USA) according to the manufacturer’s instructions. cDNA was synthesized with the High-Capacity cDNA Reverse Transcription Kit (Applied Biosystems, Foster City, CA, USA). Quantitative real-time PCR (qPCR) was performed using SYBR Green Master Mix (Applied Biosystems) on an ABI 7500 Real-Time PCR System.

Relative expression levels of collagen I and α-smooth muscle actin (α-SMA) were calculated by the ΔΔCt method ^19^, normalized to glycerol-3-phosphate dehydrogenase (G3PDH) as the housekeeping gene. Data were expressed as fold change relative to the control group. Primer sequences are presented in Table 1.

**Table 1:** Primer sequences used for SYBR Green qPCR.

Gene	Forward Primer (5′ → 3′)	Reverse Primer (5′ → 3′)
Col1a1 (Collagen type I α1)	ACCTCAGGGTATTGCTGGAC	GACCAGGGAAGCCTCTTTCT
Acta2 (α-SMA)	ACCATCGGGAATGAACGCTT	CTGTCAGCAATGCCTGGGTA
G3PDH/GAPDH (housekeeping)	GCAAGGATACTGAGAGCAAGAG	GGATGGAATTGTGAGGGAGATG

### Assessment of inflammatory cytokines levels in rat liver tissues

Tumor necrosis factor-alpha (TNF-α), total nitric oxide (NO), and interleukin-1 beta (IL-1β) levels were measured in liver homogenates. TNF-α was determined using an ELISA kit (AssayPro Co., USA; Cat. No. ERT20101) and expressed as pg/mg protein. IL-1β was quantified with a rat ELISA kit (IBL Co., Hamburg, Germany; Cat. No. RLB00) and expressed as pg/mg protein. Total NO content was assessed spectrophotometrically using vanadium trichloride and Griess reagent, as described by Miranda et al. ^20^ and expressed as nmol/mg protein.

Nuclear factor kappa B (NF-κB p65) activity was determined in nuclear extracts using an ELISA kit (Novus Biologicals, Littleton, CO, USA; Cat. No. NBP2-29,661–1) according to the manufacturer’s instructions. Results were normalized to total protein and expressed as ng/mg protein.

### Assessment of hepatic contents of lipid peroxidation and reduced glutathione

Liver homogenates were used to determine oxidative stress markers. Lipid peroxidation was assessed by measuring malondialdehyde (MDA) levels according to the method of Ohkawa et al. ^21^. Reduced glutathione (GSH) content was evaluated colorimetrically using Ellman’s reagent and glutathione reductase, following the method of Moron et al. ^22^. All values were normalized to total protein content.

### Determination of enzymatic antioxidants in rat liver

Liver homogenates were used to evaluate antioxidant enzyme activities. Superoxide dismutase (SOD) activity was measured according to the method of Marklund and Marklund ^23^. Glutathione peroxidase (GSH-Px) activity was determined by the method of Paglia and Valentine ^24^, while catalase (CAT) activity was assessed using the method of Sinha ^25^. All enzyme activities were normalized to total protein content.

### Statistical analysis

Data were expressed as mean ± SEM. Normality was assessed using the Shapiro–Wilk test, and homogeneity of variances by Levene’s test. One-way ANOVA was applied, followed by Tukey’s post hoc test for multiple comparisons. Statistical analyses and graph generation were performed using GraphPad Prism version 9.0 (GraphPad Software, San Diego, CA, USA). A *p*-value < 0.05 was considered statistically significant.

## Results

### Effects of different treatment protocol on liver state of health

**Fig. 1:**
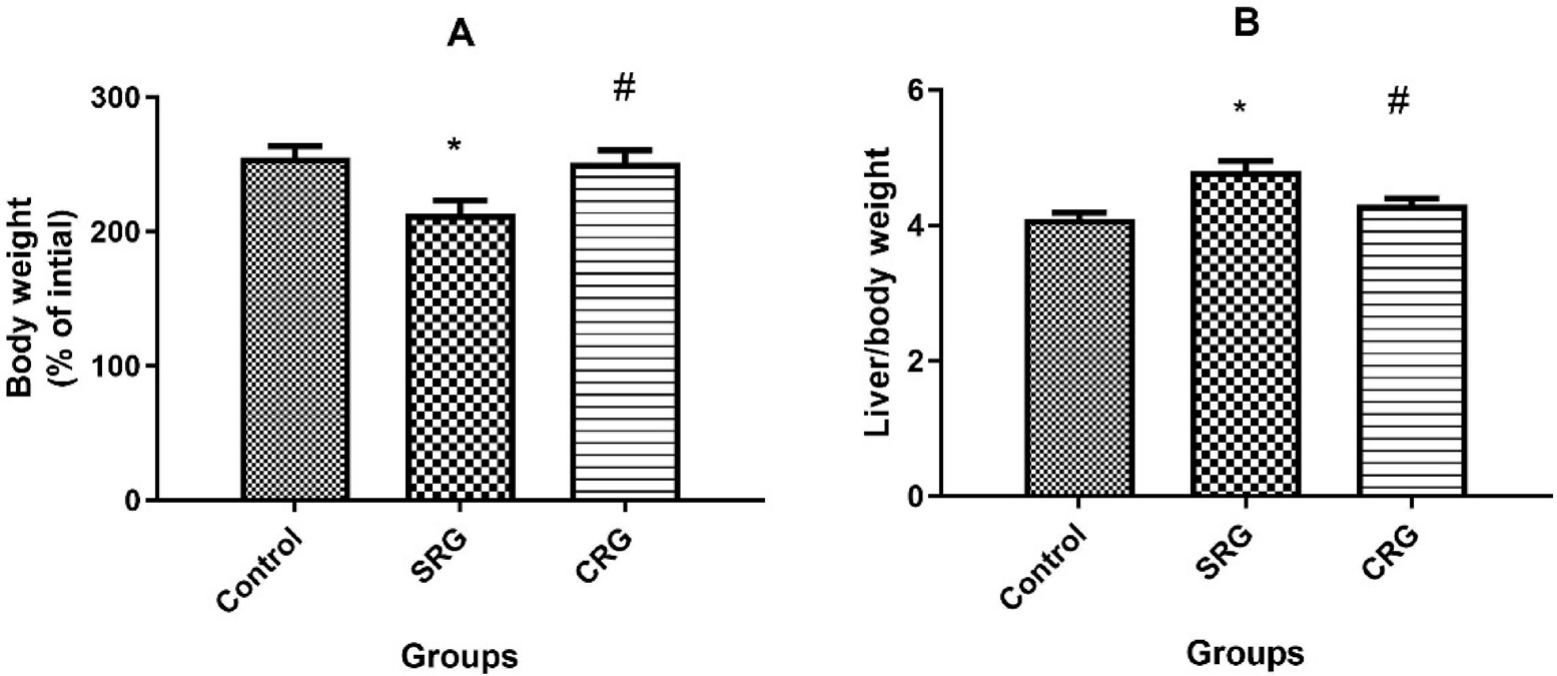
Effect of treatment on total body weight and liver weight in rats. (**A**) Body weight expressed as a percentage of the corresponding initial weight. (**B**) Relative liver weight expressed as a percentage of body weight at the end of the experiment. Animals were treated for 10 weeks (8 weeks for fibrosis induction and 2 weeks for recovery). Data are presented as mean ± SEM (n = 10 rats per group). SRG: Spontaneous recovery group; CRG: Crocin recovery group. *p ≤ 0.01 vs. Control group; #*p* ≤ 0.01 vs. SRG, using Tukey’s post hoc test following one-way ANOVA.

Liver health was assessed by evaluating body weight, the liver-to-body weight ratio, and serum biomarkers of liver integrity. Rats in the SRG group showed a significantly lower final body weight (reaching 212.8% ± 10.3 of their initial baseline weight) compared to both the control (255% ± 8.5) and CRG groups (251% ± 9.4; *p* < 0.01), with no significant difference between control and CRG. The SRG group also exhibited a significant increase in the liver-to-body weight ratio (4.8 ± 0.15%) compared to control rats (4.1 ± 0.09%; *p* < 0.01). In contrast, the CRG group showed a significantly reduced ratio (4.3 ± 0.1%; *p* < 0.01 vs. SRG), which approached control values (Fig. 1).

**Fig. 2:**
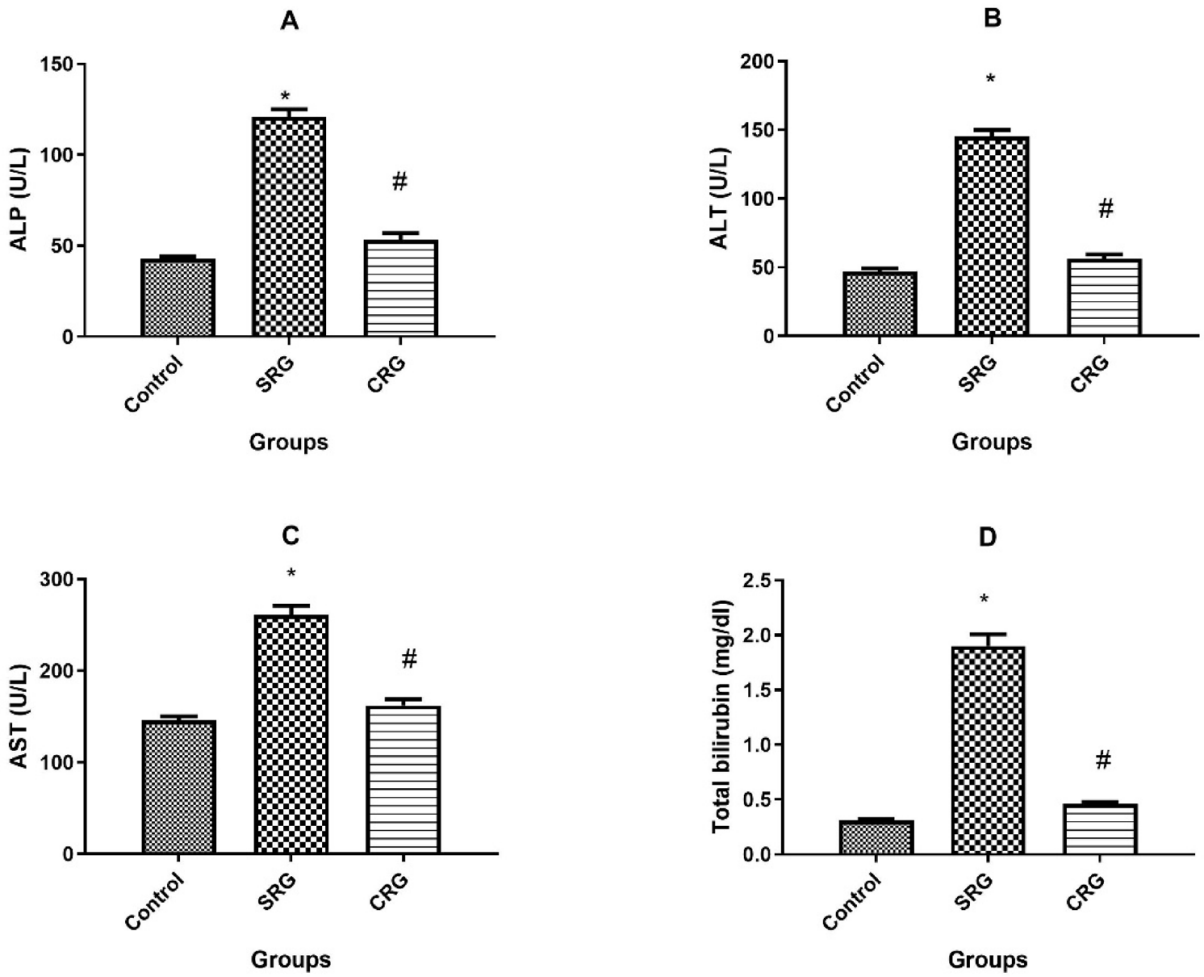
Effect of treatment protocol on serum biomarkers for liver injury in rats. (**A**) Serum alkaline phosphatase (ALP), (**B**) alanine aminotransferase (ALT), (**C**) aspartate aminotransferase (AST), and (**D**) total bilirubin levels in control, spontaneous recovery (SRG), and crocin recovery (CRG) groups. Animals were treated for 10 weeks (8 weeks for fibrosis induction and 2 weeks for recovery). Data are presented as mean ± SEM (n = 10 rats per group). *p ≤ 0.01 vs. Control group; *p* ≤ 0.01 vs. SRG, using Tukey’s post hoc test following one-way ANOVA.

Similarly, serum liver biomarkers (ALP, ALT, AST, and total bilirubin) followed the same trend. In the SRG group, all four markers were significantly elevated compared to controls (*p* < 0.01) after 14 days of CCl₄ cessation. Conversely, crocin treatment for 14 days (CRG) markedly reduced the levels of these biomarkers, showing no significant difference from control values (Fig. 2).

## Liver fibrosis state

### Serum biomarkers for liver fibrosis

**Fig. 3:**
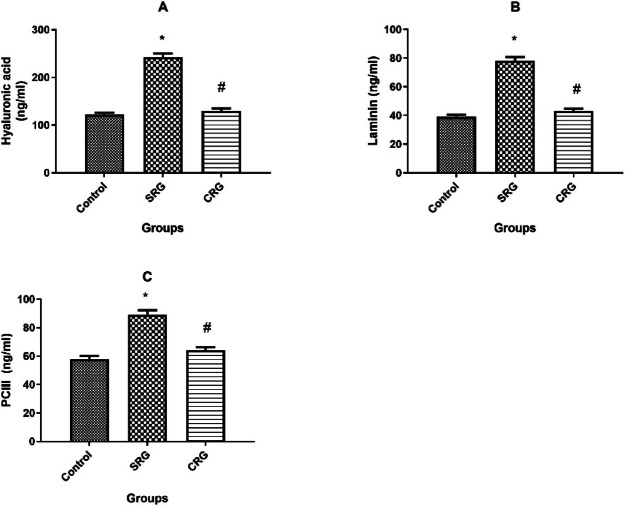
Effect of treatment protocol on serum biomarkers for liver fibrosis in rats. Serum fibrosis biomarkers in control, spontaneous recovery group (SRG), and crocin recovery group (CRG). (**A**) Hyaluronic acid, (**B**) laminin, and (**C**) procollagen type III (PCIII). Animals were treated for 10 weeks (8 weeks for fibrosis induction and 2 weeks for recovery). Data are presented as mean ± SEM (n = 10 rats per group). *p ≤ 0.01 vs. Control group; *p* ≤ 0.01 vs. SRG, using Tukey’s post hoc test following one-way ANOVA.

The SRG group showed a significant increase in serum levels of hyaluronic acid, laminin, and PCIII two weeks after CCl₄ termination (*p* < 0.01). In contrast, crocin treatment (CRG) significantly reduced these markers toward control values, with levels being markedly lower than those of SRG (Fig. 3).

### Collagen I and alpha-smooth muscle actin (α-SMA) mRNAs expressions in liver

**Fig. 4:**
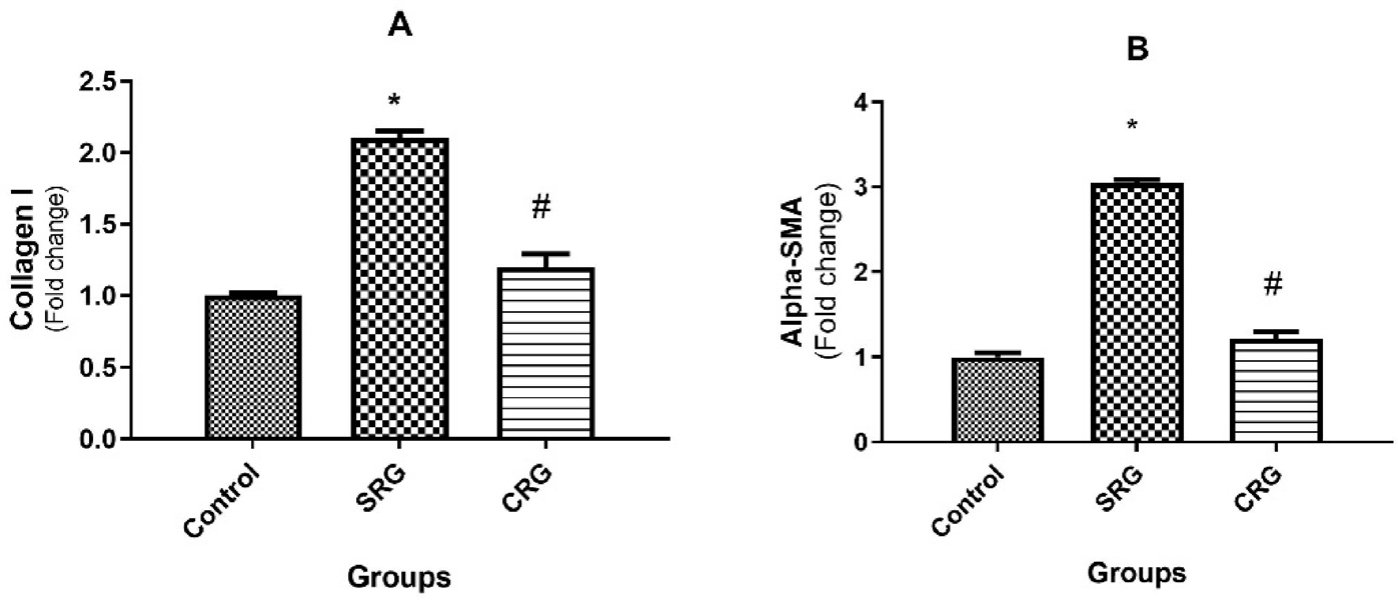
Effect of crocin on mRNA expression of collagen I and alpha-smooth muscle actin (α –SMA) in rats’ liver. Hepatic mRNA expression of fibrosis-related genes in control, spontaneous recovery group(SRG), and crocin recovery group (CRG). (**A**) Collagen I, (**B**) α-smooth muscle actin (α-SMA). Animals were treated for 10 weeks (8 weeks for fibrosis induction and 2 weeks for recovery), after which livers were collected for gene expression analysis using real-time PCR. Collagen I and α-SMA expression levels were normalized against glycerol-3-phosphate dehydrogenase (G3PDH) and presented as fold change relative to the control group. Data are presented as mean ± SEM (n = 10 rats per group). *p ≤ 0.01 vs. Control group; *p* ≤ 0.01 vs. SRG, using Tukey’s post hoc test following one-way ANOVA.

Rats in the SRG group exhibited significantly elevated mRNA expression of collagen I and α-SMA, reaching 2.1 ± 0.05- and 3.05 ± 0.04-fold of control values, respectively (*p* < 0.01). Conversely, crocin treatment for 2 weeks following CCl₄ exposure (CRG) markedly attenuated these increases. Collagen I and α-SMA expressions in CRG rats were 1.2 ± 0.09- and 1.22 ± 0.08-fold of control, respectively, which were significantly lower than SRG but still above control values (Fig. 4).

### Hepatic contents of hydroxyproline, transforming growth factor-beta (TGF- β) and tissue inhibitor of matrix metalloprotease-1 (TIMP-1)

**Fig. 5:**
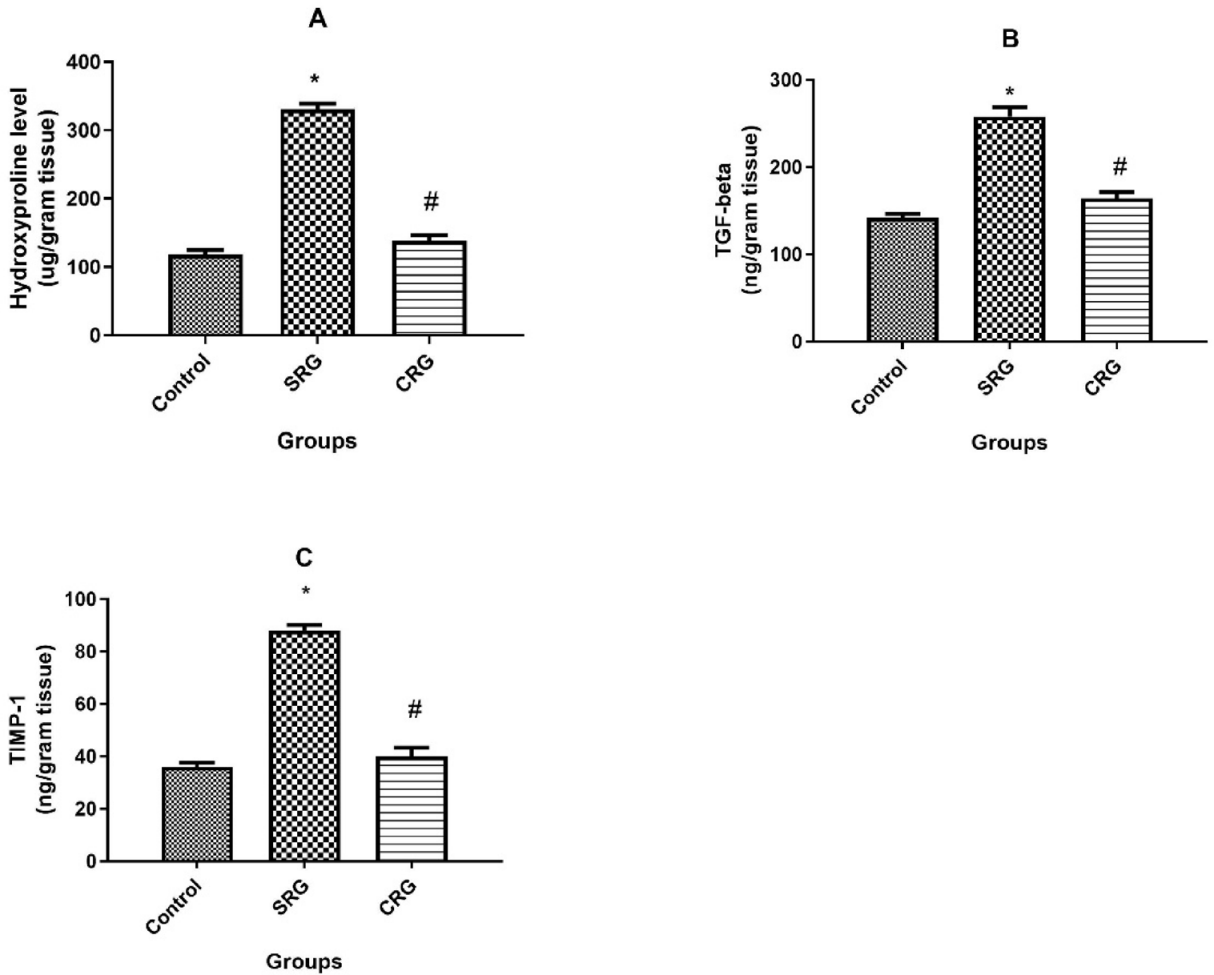
Effect of treatment on fibrosis controlling proteins contents in rats’ liver. Hepatic fibrosis-related parameters in control, spontaneous recovery group (SRG), and crocin recovery group (CRG). (**A**) Hydroxyproline content, (**B**) transforming growth factor-β (TGF-β), and (**C**) tissue inhibitor of matrix metalloproteinase-1 (TIMP-1). Animals were treated for 10 weeks (8 weeks for fibrosis induction and 2 weeks for recovery). Data are presented as mean ± SEM (n = 10 rats per group). *p ≤ 0.01 vs. Control group; *p* ≤ 0.01 vs. SRG, using Tukey’s post hoc test following one-way ANOVA.

CCl₄ administration induced significant increases in hepatic hydroxyproline, TGF-β, and TIMP-1 (331 ± 8, 258 ± 10.8, and 88 ± 2.1, respectively) compared to control values (118 ± 7, 142 ± 4.7, and 36 ± 1.6; *p* < 0.01). Crocin treatment (CRG) notably counteracted these increases, reducing hepatic hydroxyproline, TGF-β, and TIMP-1 contents to 138 ± 8.4, 164 ± 7.4, and 40 ± 3.3, respectively (Fig. 5).

### Treatment protocol modulated the level of inflammatory cytokines in rat liver

As shown in Table 2, hepatic levels of NF-κB, IL-1β, TNF-α, and total NO were significantly increased in the SRG group compared to controls (*p* < 0.01). Conversely, crocin treatment (CRG) normalized these levels, showing no significant differences from controls.

**Table 2:** Effect of treatment on liver inflammatory cytokines levels.

	NF-κB (ng/mg protein)	IL-1 beta (pg/mg protein)	TNF-α (pg/mg protein)	Total NO (nmol/mg protein)
Control	0.65 ± 0.022	35.4 ± 1.7	695 ± 12.2	0.33 ± 0.014
SRG	1.41 ± 0.04*	91 ± 3.7*	1127 ± 23.6*	0.49 ± 0.012*
CRG	0.75 ± 0.05 ^#^	42 ± 2.5^#^	713 ± 28^#^	0.37 ± 0.018^#^

*Data are presented as mean ± SEM (n = 10 rats per group). *p* ≤ 0.01 vs. Control; *p* ≤ 0.01 vs. SRG, using Tukey’s post hoc test following one-way ANOVA.

Abbreviations: SRG, spontaneous recovery group; CRG, crocin recovery group; IL-1β, interleukin-1 beta; TNF-α, tumor necrosis factor-alpha; NF-κB, nuclear factor kappa B; NO, nitric oxide.

### Treatment protocol modulated lipid peroxidation and GSH levels in rat liver

SRG rats showed a marked elevation in hepatic MDA levels (8.7 ± 0.34 nmol/mg protein) accompanied by a significant reduction in GSH content (99 ± 5.2 µmol/mg protein) compared to controls. In contrast, crocin treatment (CRG) significantly reduced MDA (4.0 ± 0.18 nmol/mg protein) and increased GSH (148 ± 7.2 µmol/mg protein) compared to SRG (Table 3).

**Table 3:** Antioxidant enzymes activities, reduced glutathione, and lipid peroxidation contents in normal liver versus fibrosis reversal.

	**CAT** (U/mg protein)	**SOD** (U/mg protein)	**GSH-PX** (U/mg protein)	**GSH** (µmol/mg protein)	**MDA** (nmol/mg protein)
Control	34 ± 2.4	351 ± 8.2	15.4 ± 0.5	136 ± 3.3	4.5 ± 0.19
SRG	21 ± 1.4*	290 ± 12*	12.7 ± 0.9*	99 ± 5.2*	8.7 ± 0.34 *
CRG	39 ± 2.1^#^	374 ± 11^#^	17.4 ± 0.7^#^	148 ± 7.2^#^	4.0 ± 0.18^#^

*Data are presented as mean ± SEM (n = 10 rats per group). *p* ≤ 0.01 vs. Control; *p* ≤ 0.01 vs. SRG, using Tukey’s post hoc test following one-way ANOVA. Abbreviations: SRG, spontaneous recovery group; CRG, crocin recovery group; CAT, catalase; SOD, superoxide dismutase; GSH-PX, glutathione peroxidase; GSH, reduced glutathione; MDA, malondialdehyde.

### Crocin treatment induced antioxidant enzymes activities in liver tissue

As illustrated in Table 3, the SRG group showed significantly reduced activities of catalase (CAT), superoxide dismutase (SOD), and glutathione peroxidase (GSH-Px) (21 ± 1.4, 290 ± 12, and 12.7 ± 0.9 U/mg protein, respectively) compared to controls (34 ± 2.4, 351 ± 8.2, and 15.4 ± 0.5; *p* < 0.01). In contrast, crocin treatment (CRG) significantly increased CAT, SOD, and GSH-Px activities relative to SRG, with values not significantly different from controls.

## Discussion

Liver fibrosis is now recognized as a dynamic and potentially reversible process once the underlying causative factors such as CCl₄, alcohol, or bile duct ligation (BDL) are removed ^2,3^. Therefore, there is a growing need for safe, effective, and affordable agents that can induce regression of fibrosis. Natural antioxidants are among the promising candidates ^2,26^. Our previous studies documented the hepatoprotective effect of crocin against acute liver injury ^17^; however, its ability to reverse established liver fibrosis had not been previously reported. Accordingly, the present study was designed to explore the curative potential of crocin in CCl₄-induced hepatic fibrosis and to investigate the possible underlying mechanisms.

The rat model of CCl₄-induced liver fibrosis was employed as it closely resembles human fibrosis in terms of pathophysiology and alterations in liver function, making it one of the most widely used models for investigating the mechanisms of fibrosis and testing novel therapeutic approaches ^8,27–29^.

In this study, CCl₄ administration induced clear manifestations of hepatic fibrosis. These included: (1) increased liver-to-body weight ratio, (2) elevated serum biomarkers of liver injury (ALP, ALT, AST, and total bilirubin) ^17^, (3) higher serum fibrosis markers (hyaluronic acid, laminin, and PCIII) ^30^, (4) upregulation of hepatic collagen I and α-SMA mRNA expression, and (5) increased hepatic hydroxyproline content ^8,31^. Collectively, these findings confirm successful induction of chronic liver injury and fibrosis in SRG rats.

Remarkably, crocin treatment for 2 weeks after CCl₄ cessation significantly reversed these pathological alterations. Crocin normalized the liver-to-body weight ratio, restored serum biomarkers of hepatic injury and fibrosis, and attenuated the expression of collagen I, α-SMA, and hepatic hydroxyproline content. These results demonstrate the ability of crocin to enhance recovery from fibrosis compared to the spontaneous recovery group (Figs. 1–5).

The therapeutic effect of crocin against CCl₄-induced fibrosis appears to be mediated by multiple mechanisms. First, crocin markedly reduced NF-κB activity and consequently lowered the hepatic levels of pro-inflammatory cytokines including TNF-α, IL-1β, and total NO, thereby attenuating the inflammatory response associated with chronic liver injury. Second, crocin suppressed profibrogenic mediators such as TGF-β and TIMP-1, leading to reduced extracellular matrix (ECM) deposition, as evidenced by decreased collagen I and α-SMA expression together with lower hydroxyproline content. Third, crocin alleviated oxidative stress, as demonstrated by reduced MDA levels, increased hepatic GSH content, and enhanced antioxidant enzyme activities (CAT, SOD, and GSH-Px). Collectively, these data indicate that the antifibrotic action of crocin is attributable to a combination of anti-inflammatory, antifibrotic, and antioxidant properties.

Our findings align with previous reports demonstrating the reversibility of hepatic fibrosis, particularly at early stages where extracellular matrix (ECM) crosslinking and angiogenesis are limited ^3,15,32,33^. Spontaneous recovery after CCl₄ withdrawal has been attributed to mechanisms such as ECM degradation, apoptosis of activated hepatic stellate cells (HSCs), reduction of inflammatory and fibrogenic cytokines, and activation of metalloproteinases, particularly collagenases ^3,6,32,34^. Matrix metalloproteinases (MMPs) are key regulators of ECM degradation; however, their activity is suppressed by TIMPs. TIMP-1 not only inhibits MMPs but also protects activated HSCs from apoptosis^32,35,36^. In agreement with these observations, crocin treatment in our study markedly reduced hepatic TIMP-1 levels, which may have facilitated ECM resolution.

Similarly, NF-κB has been recognized as a central mediator of both inflammation and fibrosis ^8,37–40^. It regulates the expression of pro-inflammatory cytokines (TNF-α, IL-1β, NO, and TGF-β), creating a positive feedback loop that amplifies chronic inflammation ^37–41^. Our data confirm this pathway, as CCl₄ exposure markedly increased NF-κB and downstream cytokines, while crocin administration attenuated these responses.

Furthermore, oxidative stress has been identified as a critical inducer of NF-κB activation in liver fibrosis ^27,42,43^. Consistently, crocin, with its strong antioxidant capacity, effectively abrogated CCl₄-induced oxidative stress, as reflected by reduced MDA and increased GSH levels together with enhanced antioxidant enzyme activities. This antioxidant action likely contributed to the observed suppression of NF-κB and pro-inflammatory cytokines ^43,44^. Our findings therefore support previous evidence that antioxidants can mitigate hepatic fibrosis ^28,31,44–46^, while extending this concept by demonstrating, for the first time, the curative potential of crocin in an established fibrosis model.

A limitation of the present study is the absence of histopathological or immunohistochemical confirmation (e.g., H&E, Masson’s trichrome, or α-SMA staining), which are commonly employed to visualize structural changes during fibrosis. Nevertheless, we compensated for this limitation by employing a comprehensive panel of biochemical and molecular markers, including serum fibrosis biomarkers (hyaluronic acid, laminin, PCIII), hepatic hydroxyproline, TGF-β, TIMP-1, inflammatory cytokines, and the mRNA expression of collagen I and α-SMA. Together, these multi-level assessments provide robust and reliable evidence of fibrosis progression and recovery. Future studies should integrate histological and immunohistochemical analyses, as well as different models of liver fibrosis and longer treatment durations, to further validate the therapeutic potential of crocin.

Overall, our findings highlight crocin as a promising natural compound capable of reversing CCl₄-induced hepatic fibrosis in rats. By targeting interconnected pathways of oxidative stress, inflammation, and fibrogenesis, crocin significantly enhanced recovery beyond spontaneous regression. These results provide a solid experimental basis for considering crocin as a potential antifibrotic therapy, while emphasizing the need for further studies incorporating histopathological confirmation, extended treatment durations, and translational validation.

## Conclusion

This study provides clear evidence for the curative potential of crocin in recent CCl₄-induced hepatic fibrosis in rats. The therapeutic effects of crocin appear to be mediated through attenuation of oxidative stress, suppression of NF-κB signaling and its downstream inflammatory cytokines (IL-1β, TNF-α, and NO), and reduction of profibrogenic mediators such as TIMP-1 and TGF-β, ultimately leading to diminished collagen accumulation in hepatic tissue. Taken together, our findings highlight crocin as a safe and promising natural antioxidant with potential curative utility against liver fibrosis in rats. Future studies using histopathological confirmation, multiple models of liver fibrosis, longer treatment durations, and graded doses are warranted to further validate these results and explore their translational relevance.

## Data Availability

The datasets supporting the conclusions of this article are included within the article. Any further details about the data and materials of this study are transparent and available from the corresponding author upon reasonable request.

## Abbreviations

ALP: Alkaline phosphatase.
ALT: Alanine aminotransferase.
AST: Aspartate aminotransferase.
CAT: Catalase.
CCl4: Carbon tetrachloride.
CRG: Crocin recovery group.
ECM: Extracellular matrix.
G3PDH: Glycerol-3-phosphate dehydrogenase.
GSH: Reduced glutathione.
GSH-Px: Glutathione peroxidase.
HSCs: Activated hepatic stellate cells.
IL 1β: Interleukin-l beta.
MDA: Malondialdehyde.
NF-κB: Nuclear factor kappa-light-chain-enhancer of activated B cells.
NO: Total nitric oxide.
ROS: Reactive oxygen species.
SOD: Superoxide dismutase.
SRG: Spontaneous recovery group.
TGF-β: Transforming growth factor beta.
TIMP-1: Tissue inhibitor of matrix metalloproteinase-1.
TNF-α: Tumor necrosis factor-alpha.
*α* –SMA: Alpha smooth muscle actin
